# Melatonin Ameliorates Age‐Related Sarcopenia via the Gut–Muscle Axis Mediated by Serum Lipopolysaccharide and Metabolites

**DOI:** 10.1002/jcsm.13722

**Published:** 2025-02-03

**Authors:** Ling‐Shan Zhou, Yuan Yang, Li Mou, Xin Xia, Min Liu, Ling‐Jie Xu, Rong Liu, Jun‐Ping Liu, Hai‐Yan Zhang, Xiao‐Jun Ao, Chang‐Jiang Liu, Qian Xiao, Shi‐Xiong Liu

**Affiliations:** ^1^ Department of Geriatrics Ward 2 The First Hospital of Lanzhou University Lanzhou China; ^2^ Department of Gastroenterology The First Affiliated Hospital, Hengyang Medical School, University of South China Hengyang China; ^3^ NHC Key Laboratory of Birth Defects and Reproductive Health Chongqing Population and Family Planning Science and Technology Research Institute Chongqing China; ^4^ The Center of Gerontology and Geriatrics and National Clinical Research Center for Geriatrics West China Hospital of Sichuan University Chengdu China; ^5^ Department of Gastroenterology The First Hospital of Lanzhou University Lanzhou China; ^6^ Department of General Practice The First Affiliated Hospital of Chongqing Medical University Chongqing China; ^7^ Department of Rehabilitation The First Hospital of Lanzhou University Lanzhou China; ^8^ Department of Clinical Nutrition The First Hospital of Lanzhou University Lanzhou China; ^9^ The Second Affiliated Clinical School Guangzhou University of Chinese Medicine Guangzhou China; ^10^ Department of Geriatrics The First Affiliated Hospital of Chongqing Medical University Chongqing China

**Keywords:** gut–muscle axis, LPS, mediation analysis, melatonin, metabolites, sarcopenia

## Abstract

**Background:**

Sarcopenia affects the quality of life and increases adverse outcomes in the elderly. However, as a potential safe and effective remedy to many age‐related disorders, little is known about the protective effect of melatonin against sarcopenia, especially the underlying mechanisms of pathophysiology related to the gut–muscle axis.

**Methods:**

The young (4 months) and old‐aged (24 months) wild‐type C57BL/6J male mice were included in this study, of which the old‐aged mice in the experimental group were treated with 10 mg/kg/day of melatonin for 16 weeks. After that, muscle strength, muscle mass and the cross‐sectional area (CSA) of the gastrocnemius muscle fibres were measured. Then, the putative pathways, based on the data obtained from 16S rDNA sequencing of the gut microbiota, RNA sequencing of gastrocnemius muscle and serum untargeted metabolomics, were screened out by the integrated multiomics analysis and validated using immunohistochemistry, ELISA and TUNEL staining. C2C12 myoblasts were treated with LPS. Flow cytometric analysis and western blotting were applied to detect cell apoptosis and protein expressions of Tnfrsf12a and caspase8, respectively. In addition, the mediation analysis was carried out to infer the causal role of the microbiome in contributing to the skeletal muscle through metabolites.

**Results:**

Melatonin treatment ameliorated age‐related declines in muscle strength (*p* < 0.05), muscle mass (*p* < 0.01) and CSA of the gastrocnemius muscle fibres (*p* < 0.01), as well as changed the gut microbial composition (beta‐diversity analysis; *R* = 0.513, *p* = 0.005). The integrated multiomics analysis implied two main mechanisms about the impact of melatonin‐related modifications in the gut microbiota on sarcopenia. First, a lower serum lipopolysaccharide (LPS) level associated with the altered gut microbiota was observed in melatonin‐treated mice (*p* < 0.001) and was most relevant to the transcription level of Tnfrsf12a in skeletal muscle (*R* = 0.926, *p* < 0.001). Further bioinformatics analyses and in vitro experiments showed that LPS could contribute to skeletal muscle apoptosis by regulating the Tnfrsf12a/caspase‐8 signalling pathway. Second, melatonin significantly altered serum metabolites (variable importance on projection (VIP) > 1.5, *p* < 0.05). Mediation models showed that changes in the gut microbiome also influenced skeletal muscle through these metabolites (27 linkages; BH‐adjusted *p* < 0.05).

**Conclusions:**

Our study revealed functional insights and a putative causality for the role of the gut–muscle axis in the mechanism of melatonin ameliorating age‐related sarcopenia, namely, inhibition of the LPS‐induced Tnfrsf12a/caspase‐8 signalling pathway or serum metabolites as intermediates in the gut–muscle axis.

## 1 Introduction

Aging inevitably influences the structure and function of skeletal muscle, leading to the progressive decrease in both muscle strength and quality in the elderly [1]. Sarcopenia, as the most common indicator of skeletal muscle ageing, is the deterioration in skeletal muscle quality and functionality. Sarcopenia has attracted significant worldwide attention, as it affects the quality of life and increases adverse outcomes including falls, fractures, frailty and mortality [2, 3]. As the population ages, sarcopenia has become a major challenge, with consequences at the individual and societal levels. Despite the profound impact, the tools for preventing and treating sarcopenia are limited, with only exercise and nutrition available so far.

Melatonin, a biological hormone normally produced in all organisms, is a highly effective antioxidant, free radical scavenger, immune modulator and endocrine regulator [4]. Together with the fact that melatonin progressively declines with increasing age, it is suggested that melatonin is related to the incidence or severity of age‐associated diseases, such as Parkinson's disease and Alzheimer's disease (Supporting Information: References S1 and S2). The compelling evidence of melatonin slowing down ageing and counteracting age‐related disorders has sparked research on its effects on sarcopenia. In postmenopausal women, there was an inverse association between urine melatonin and sarcopenia [5]. Oral melatonin administration reduced age‐mediated signs of sarcopenia [6]. In vitro experiments also exhibited beneficial or promising actions of melatonin for sarcopenia, such as the decrease in oxidative stress and mitochondrial damage, alteration in autophagy, improvement in protein synthesis, increase in satellite cells and decrease in apoptosis and inflammation [7]. These findings of the research suggest that melatonin has excellent application prospects for preventing sarcopenia, especially in age‐related sarcopenia. To date, however, the role of melatonin in ageing skeletal muscles is still poorly understood, and its mechanisms need further in‐depth research.

Thanks to the high‐throughput molecular techniques, the direct or indirect role of the gut microbiota in host physiology and pathophysiology is being increasingly recognized, revealing an intimate crosstalk between the gut microbiota and other organs, such as the discovery of the gut‐liver axis and gut‐brain axis (Supporting Information: References S3 and S4). Because of the extensive influence on health, the gut microbiota has been considered as a ‘central organ’ [8]. In the past few years, the novel concept of the ‘gut–muscle axis’ has been proposed based on the current available evidence on how the gut microbiota may affect skeletal muscle mass and metabolism [9, 10]. And the mechanisms of the gut–muscle axis have intrigued researchers from diverse scientific fields including ageing. In the process of ageing, not only skeletal muscle, but also gut microbiome undergoes a series of changes, namely age‐related gut dysbiosis, with the altered biological diversity, stability and function of the gut microbiota [11]. And the gut dysbiosis may contribute to age‐related changes in skeletal muscle mass and function [12]. A recent study demonstrated that the *Lactobacillus casei* Shirota supplementation was able to attenuate age‐related sarcopenia in senescence‐accelerated mouse prone‐8 (SAMP8) mice [13]. Thus, emerging evidence has related the gut–muscle axis to the onset and progression of age‐related sarcopenia. The melatonin supplementation could ameliorate HFD‐induced gut microbiota dysbiosis as a probiotic agent [14]. What is more, melatonin could reverse the sleep deprivation‐induced cognitive impairment by gut microbes and their metabolites [15]. These findings demonstrate the effect of melatonin on regulating a crosstalk between the gut microbiota and other organs. To date, however, very little is understood about the underlying mechanisms by which melatonin improves the pathophysiology of age‐related sarcopenia, especially its role in the gut–muscle axis.

In this study, we performed integrated omics analyses and mediation models to reveal functional insights and a putative causality for the role of the gut–muscle axis in the mechanism of melatonin ameliorating age‐related sarcopenia. Firstly, the effect of melatonin on reversing sarcopenia was verified in naturally ageing mice. The previous studies have indicated that age‐related gut dysbiosis may contribute to sarcopenia [12]. Thus, we further explored the changes in gut microbial composition after melatonin treatment. Based on the predicted function of gut microbiota modified by melatonin, the Tnfrsf12a/caspase‐8 signalling pathway mediated by gut microbiota‐derived LPS was explored. Secondly, we profiled serum metabolites and used the mediation analysis to assess whether they serve as intermediates in gut–muscle communication.

## 2 Methods

### 2.1 Animal Treatment Protocol for the Study

Wild‐type C57BL/6J male mice (8 weeks old) were purchased from SPF Biotechnology Co., Ltd. (Beijing, China) and housed in Chongqing Population and Family Planning Science and Technology Research Institute individually with controlled temperature (22°C ± 1°C), humidity (40%–60%) and 12‐h light/dark cycle. Each mouse had free access to water and standard solid food. The feed composition and nutritional information were shown in the Supporting Information.

Eighteen mice were divided into the following three groups: mature young mice (Y, 4 months), old‐aged mice (Con, 24 months) and old‐aged mice with melatonin (Mel, 24 months). Starting at the age of 20 months, the mice in the Mel group were treated with water supplemented with melatonin (M5250, MCE, USA). The dosage of melatonin was 10 mg/kg/day during the 16 weeks of treatment, and the concentration was adjusted based on the body weight and daily water consumption. Melatonin was protected from the light by covering with aluminium foil on drinking water bottles.

### 2.2 Grip Strength Test

Muscle strength was evaluated by measuring the peak of forelimb grip strength using a gripping force instrument (SA415, Sansbio, China). The mouse was lifted and placed so that the forelimbs grasped the sensing cross bar. After that, the tail was dragged backward with the body horizontal until the forelimb was released. The grip force instrument automatically recorded the maximum grip strength of mice in this process. Each mouse was tested three times, and the maximum value was recorded and included in the further calculation.

### 2.3 Tissue Collection

Following completion of the intervention, mice were weighed, anaesthetized with 5% chloral hydrate and then sacrificed. Blood was collected by cardiac puncture. Gastrocnemius muscles were isolated from both hind limbs and weighed. Muscle samples for RNA‐Seq were immediately frozen in liquid nitrogen. Muscle samples for histology were fixed in 4% paraformaldehyde at room temperature, dehydrated with a gradient ethanol series and then embedded in paraffin.

### 2.4 Haematoxylin and Eosin Staining

Embedded muscle tissue blocks were cut to obtain 4 μm transverse tissue sections. After dewaxing, slides were dehydrated in ethanol series with ascending graded concentrations. Then, they were stained with haematoxylin and eosin. Images were acquired using a Motic digital imaging system (EasyScan 6, Motic, China), and the average cross‐sectional area (CSA) of the myofibers was calculated using the Image‐Pro Plus software.

### 2.5 RNA‐Seq Analysis

RNA‐Seq was performed as described in Methods S1.

### 2.6 16S rDNA Gene Sequencing and Data Analysis

Faecal samples were collected with the instruments sterilized and immediately stored at −80°C until further processing. The detailed experimental methods and data analyses were presented in Methods S2.

### 2.7 Untargeted Metabolomics Analysis

Serum samples were collected and mixed with methanol to precipitate proteins. Then the obtained supernatant was removed and used for the liquid chromatography‐mass spectrometry (LC‐MS) analysis. The detailed experimental methods and data analyses were presented in Methods S3.

### 2.8 Immunohistochemistry

The content of Tnfrsf12a and caspase‐8 proteins in gastrocnemius muscles was examined using immunohistochemical staining. The slides were dewaxed and hydrated, followed by retrieving antigens with a citric acid buffer and inactivating endogenous peroxidase with 3% hydrogen peroxide. After blocking with 10% normal goat serum and 1% bovine serum albumin (BSA), the slides were incubated with primary antibodies against Tnfrsf12a (1:200 dilution; ab109365, Abcam, USA) and caspase‐8 (1:200 dilution; YM3377, Immunoway, USA). Subsequently, the slides were washed with PBS and then incubated with the secondary antibody. Lastly, colour development of the slides was performed by DAB solution and pictures were captured with a Motic digital imaging system (EasyScan 6, Motic, China).

### 2.9 Terminal Deoxynucleotidyl Transferase dUTP Nick End Labelling (TUNEL) Staining

After deparaffinization and rehydration in a gradient ethanol series, antigen retrieval was performed on the muscle sections. Subsequently, each tissue was permeabilized with 0.1% Triton X‐100. In a wet box, these sections were incubated with the TUNEL reaction reagents from the TUNEL kit (40306ES20, Yeasen, China) at 37°C for 1 h. After washing with PBS for three times, the muscle sections were incubated with the 4′,6‐diamidino‐2‐phenylindole (DAPI) solution at 37°C for 10 min in the dark. Positive expressions were observed and imaged using fluorescence microscopy. The ratio of apoptotic nuclei (green light) to DAPI‐stained nuclei (blue light) was calculated as the percentage of apoptosis.

### 2.10 Lipopolysaccharides (LPS) Quantification

The concentrations of LPS in serum and faeces were determined using lipopolysaccharides (LPS) ELISA kits (ml022423, MLBIO, China) according to the manufacturer's protocols.

### 2.11 Cell Culture and Treatment

C2C12 myoblasts were purchased from Shanghai Zhong Qiao Xin Zhou Biotechnology Co., Ltd (Shanghai, China). These cells were cultured in Dulbecco's modified Eagle's medium (DMEM) containing 10% foetal bovine serum, penicillin (100 U/mL) and streptomycin (100 mg/mL) in a humidified incubator with 5% CO_2_ at 37°C. To investigate the direct effects of LPS, C2C12 myoblasts were treated with LPS (10 μg/mL) for 24 h. This duration and concentration of LPS was shown to be efficient for inducing the changes of cell fate of C2C12 myoblasts (Supporting Information: Reference S5). In some experiments, C2C12 myoblasts were transfected with Tnfrsf12a siRNA (Genepharma, China) before LPS stimulation. The cells were transfected small interference RNA (si‐Tnfrsf12a) targeting Tnfrsf12a (Forward 5′‐UCGACAAGUGCAUGGACUGTT‐3′, and Reverse 5′‐CAGUCCAUGCACUUGUCGATT‐3′) for 48 h using Lipofectamine 2000 (Invitrogen, USA) according to the manufacturer's instructions.

### 2.12 Western Blot

Total proteins were extracted using RIPA buffer supplemented with protease inhibitor (ThermoFisher Scientific, American). Protein concentration was determined using the BCA protein Assay kit (Solarbio, Beijing, China). After being boiled at 95°C for 10 min, protein samples were isolated by SDS‐polyacrylamide gel electrophoresis (SDS‐PAGE) and then transferred to polyvinylidene fluoride (PVDF) membranes (Millipore). The membranes were incubated with primary antibodies against Tnfrsf12a (ab109365, Abcam, USA) and Caspase 8 (YM3377, Immunoway, USA), followed by a secondary antibody for 1 h. Membranes were developed with ECL substrate (Beyotime Biotechnology, Shanghai, China) and exposed using film.

### 2.13 Flow Cytometry

Apoptosis in C2C12 cells was assessed by Annexin V‐FITC and propidium iodide (PI) double staining kits (40302ES, Yesen, China) according to the manufacturer's instructions. Briefly, C2C12 cells were harvested and resuspended in annexin binding buffer. Afterwards, cells were stained with Annexin V‐FITC and PI for 15 min on ice. The percentage of apoptotic cells was measured by a LSR II Flow Cytometer (BD Biosciences).

### 2.14 Mediation Analysis

To further infer the causal role of the microbiome in contributing to muscle transcriptomic changes through metabolites, the mediation analysis was conducted using the R package ‘mediation’. The mediation analysis is based on the counterfactual framework and offers a way to explain the mechanism by which an exposure affects an outcome [16]. In this study, it not only assessed whether there actually was a causal relationship between the exposure ‘microbiota’ and the outcome ‘DEGs’, but also focused on how the relationship was mediated by the factors, namely whether metabolites acted as the mediator for the effect of microbiome on DEGs. In this model, the direct, indirect and total causal effects for each subject were estimated by computing the coefficients of linear regression eqs. Subsequently, the causal effects were averaged to get the average causal effects, including average causal mediation effects (ACME), average direct effects (ADE) and total effects. And the proportion mediated (Prop) represented the ratio of the indirect effect estimate to the total effect estimate. The statistically significant results with BH‐adjusted *p*_ACME_ < 0.05, BH‐adjusted *p*_Prop_ < 0.05, BH‐adjusted *p*_ADE (microbiota‐metabolites)_ < 0.05 and BH‐adjusted *p*_ADE (metabolites‐DEGs)._ < 0.05, were shown in the Sankey diagram.

### 2.15 Statistical Analysis

Data were processed and analysed using the R version 4.0.2 or Graph‐pad Prism V.8 software and were expressed as mean values ± standard deviation (SD). Differences between three groups were analysed using ANOVA. Differences between two groups were analysed using the Student's *t*‐test or Mann–Whitney *U* test as appropriate. Based on the OTUs generated by 16S rDNA sequencing, serum metabolites and DEGs, a Pearson correlation analysis and a Mantel‐test analysis were conducted by the R package ‘ggcor’. *p* < 0.05 was considered statistically significant.

## 3 Results

### 3.1 Melatonin Prevents the Progression of Sarcopenia in Aged Mice

**FIGURE 1:**
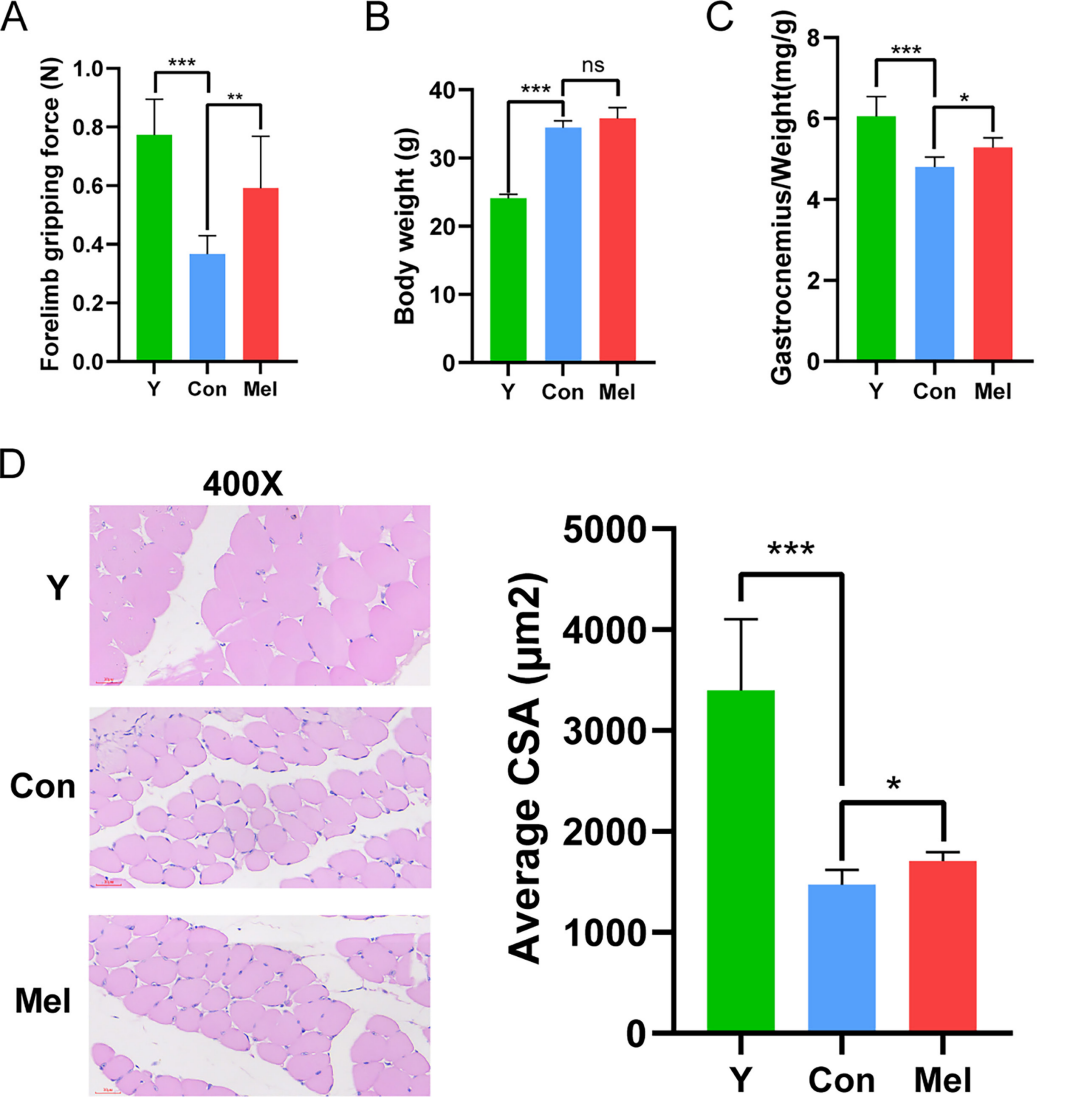
Treatment with melatonin prevents the progression of sarcopenia in aged mice. (A) Treatment with melatonin ameliorates the age‐related loss of grip strength in aged mice. (B) Treatment with melatonin has no effect on weight in aged mice. (C) Treatment with melatonin alleviates the age‐related decrease in the ratio of gastrocnemius weight to body weight. (D) Treatment with melatonin relieves the age‐related decline in CSA of the muscle fibres. *p < 0.05, **p < 0.01, ***p < 0.001, and ns = nonsignificant, Y versus Con or Con versus Mel. *n* = 6.

The diagnosis and evaluation of sarcopenia are mainly based on muscle strength and muscle quality. We first evaluated whether the naturally ageing mouse models used in this study met the criteria of sarcopenia. As expected, the forelimb grip strength of aged mice was significantly reduced compared with that of the young mice (Figure 1A). The gastrocnemius, as the muscle most affected by ageing in wild‐type mice, was often used as the target muscle of sarcopenic mouse models in previous studies. And the ratio of gastrocnemius weight to body weight in mouse models is considered as a diagnostic indicator of sarcopenia. As shown in Figure 1B, the body weight of the young mice was 24.12 ± 0.58 g, whereas the aged mice reached 34.43 ± 1.04 g. Similar to muscle strength, the ratio of gastrocnemius weight to body weight in the aged group was significantly decreased compared with that in the young group (Figure 1C). In addition, the average CSA of the gastrocnemius muscle fibres was also significantly reduced in the aged group (Figure 1D). It could be concluded that the naturally ageing mouse model used in this study was suitable for the study of sarcopenia. Subsequently, we evaluated the effect of melatonin on the phenotypes of sarcopenia. The results displayed that melatonin administration led to a significant elevation in forelimb grip strength and the ratio of gastrocnemius weight to body weight of aged mice (Figure 1A,C). On the other hand, the CSA of the gastrocnemius muscle fibres was also improved by the supplementation with melatonin. The aged mice in the melatonin‐treated group had larger muscle fibre sizes compared with the aged mice in the control group (Figure 1D). Overall, these data preliminarily confirm that melatonin could protect against age‐related declines in muscle strength and muscle quality.

### 3.2 Effects of Melatonin on the Gut Microbial Composition in Aged Mice

**FIGURE 2:**
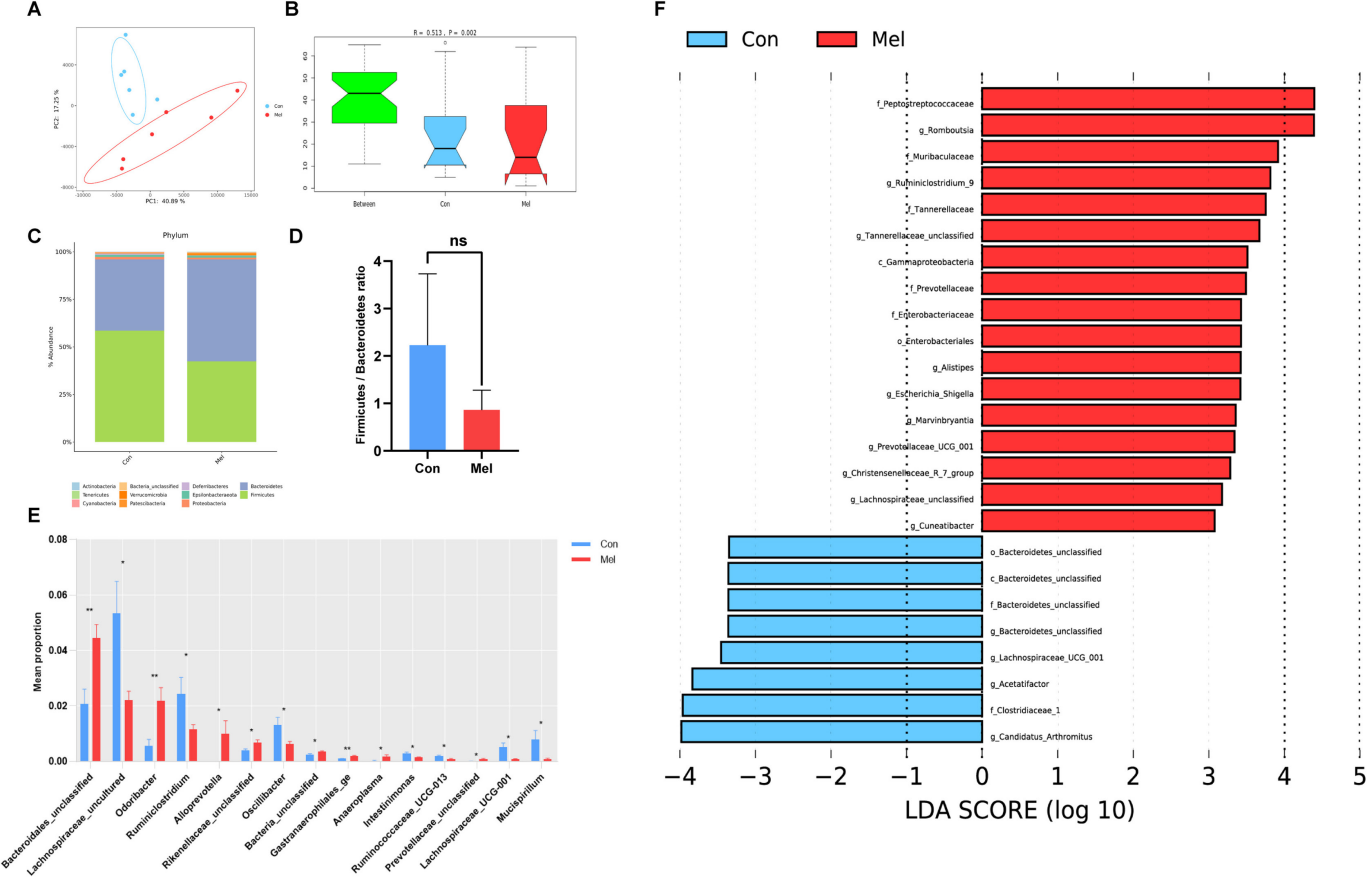
Treatment with melatonin modifies the composition of the gut microbiome. (A) Principal component analysis (PCA) of intestinal microbiome; (B) analysis of similarities (ANOSIM) of intestinal microbiome; (C) intestinal microbe relative abundance at the phylum level; (D) the ratio of *Firmicutes* to *Bacteroidetes* in intestinal microbiome at the phylum level; (E) significant changes in abundance of intestinal microbe at the genus levels; (F) line discriminant analysis (LDA) effect size (LEfSe) analysis. *p < 0.05, **p < 0.01, and ns = nonsignificant, Con versus Mel. *n* = 6.

To examine the impact of melatonin on the gut microbiota in aged mice, we investigated the microbial community and diversity by 16S rRNA sequencing technology. The clean sequencing reads, with at least 109 195 reads per sample, were obtained for the study. The results showed no significant differences in alpha‐diversity of intestinal microbiota between two groups (Figure S1A,B). However, we observed a significant variation in beta‐diversity compared with the control group (Figure 2A). And the difference in microbial species composition was larger between groups than within groups (Figure 2B). These results indicated that melatonin altered the intestinal microbial species composition but not abundance within communities in aged mice. We further analysed intestinal microbiota composition from phylum to genus. At the phylum level, *Firmicutes and Bacteroidetes* accounted for more than 95% (Figure 2C). Compared with the control, the proportion of *Bacteroidetes* was increased by 16.11%, whereas the proportion of *Firmicutes* was decreased by 16.15%. However, the ratio of *Firmicutes*/*Bacteroidetes* was not different (*p* = 0.058, Figure 2D). At the genus level, *Muribaculaceae_ge* and *Lachnospiraceae_NK4A136* were dominant genera (Figure S1C). Melatomin treatment increased the abundance of *Bacteroidales_un*, *Odoribacter* and *Gastranaerophilales_ge* (Figure 2E, Table S1). We analysed the differences in intestinal microbiota structure between two groups by LEfSe analyses, which emphasized statistical and biological significance. As shown in the histogram of LDA scores, the dominant bacteria of aged mice given melatonin were *Peptostreptococcaceae*, *Romboutsia*, *Muribaculaceae*, *Ruminiclostridium_9*, *Tannerellaceae* and so on (Figure 2F). So, these dominant bacteria could be potential biomarkers for melatonin to alleviate sarcopenia.

Next, we predicted the functions of the microbiota and observed significantly different KEGG pathway abundance in other glycan degradation, ubiquinone and other terpenoid‐quinone biosynthesis, lipopolysaccharide (LPS) biosynthesis, phosphonate and phosphinate metabolism, protein digestion and absorption, steroid hormone biosynthesis, linoleic acid metabolism, geraniol degradation and nitrotoluene degradation (Table S2). The result of microbial co‐occurrence network showed that *Bacteroidetes* and *Firmicutes* were the dominant species that dominate interactions in the intestinal environment with and without given melatonin, respectively (Figure S1D,E). Interestingly, compared with the control, there existed a higher density of interactions of microbiota in this environment given melatonin, which revealed that melatonin altered the interconnection patterns between intestinal microbiomes in aged mice.

### 3.3 Transcriptomics Reveals the Mechanism of Melatonin Action in Skeletal Muscle

**FIGURE 3:**
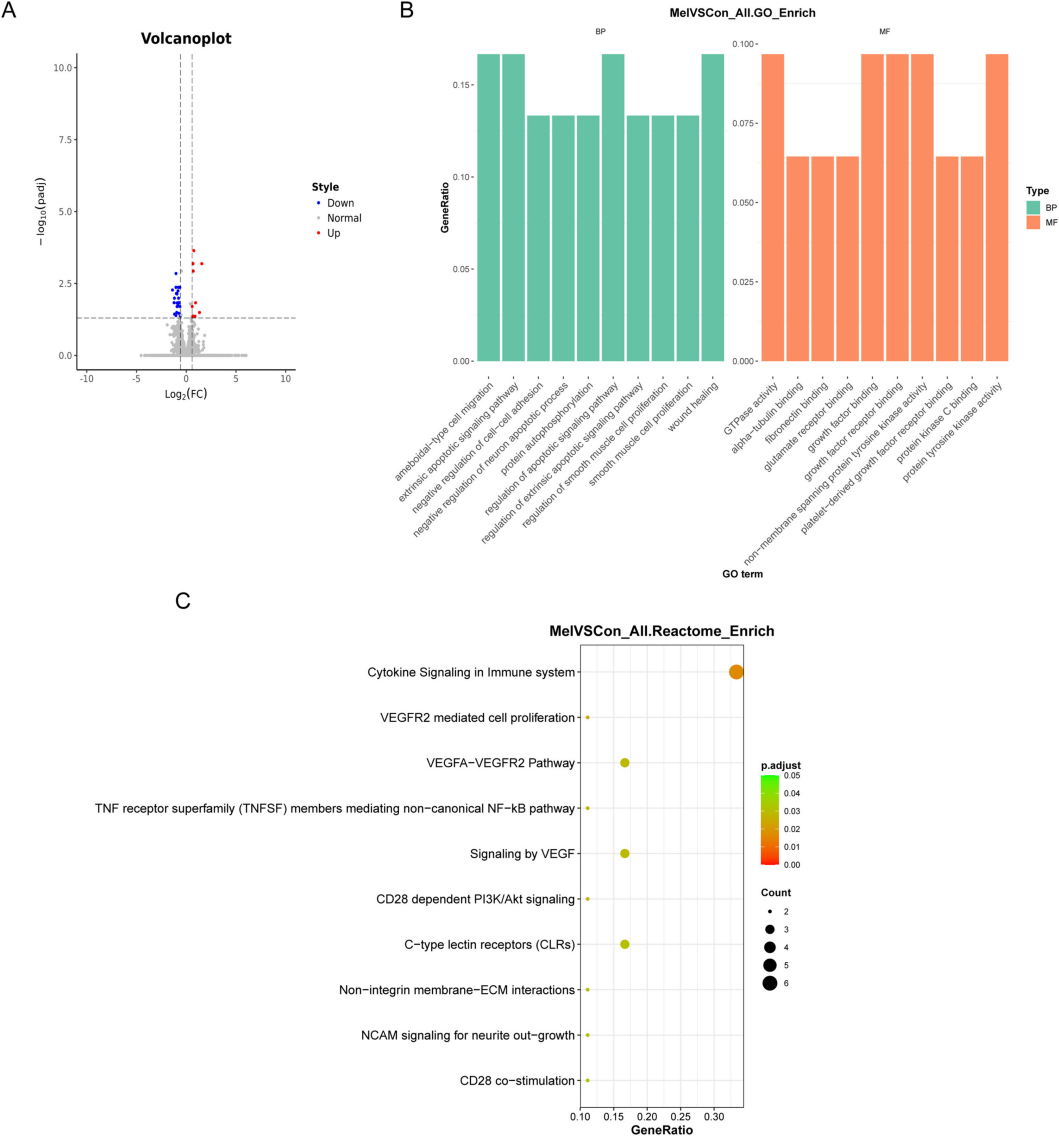
Treatment with melatonin has an effect on the transcriptomics of skeletal muscle in aged mice. (A) The volcano plot for differentially expressed genes (DEGs) in skeletal muscle; (B) significantly enriched biological processes and molecular function in GO; (C) significantly enriched pathways in Reactome. *n* = 6.

To elucidate the mechanism of melatonin in ameliorating sarcopenia, we further investigated the impact of melatonin on the transcriptome of gastrocnemius. A total of 33 DEGs were identified, including 9 genes were upregulated and 24 genes were downregulated (Figure 3A, Table S3). Then, we explored the biological characteristics of these DEGs by performing GO and Reactome analyses. Our results showed that these DEGs were mainly enriched in the apoptosis‐related biologic process and pathways, such as the extrinsic apoptotic signalling pathway, extrinsic apoptotic signalling pathway regulation, apoptotic signalling pathway regulation, cytokine signalling of the immune system and noncanonical NF‐κB pathways mediated by TNF receptor superfamily members (Figure 3B,C). These results suggest that the identified DEGs are possibly involved in skeletal muscle apoptosis, and these related processes and pathways may be the potential mechanisms by which melatonin alleviates age‐related sarcopenia.

### 3.4 The Tnfrsf12a/Caspase‐8 Signalling Pathway Is Involved in the Mechanism by Which LPS Derived From the Gut Microbiota Induced Apoptosis in Sarcopenia via the Gut–Muscle Axis

**FIGURE 4:**
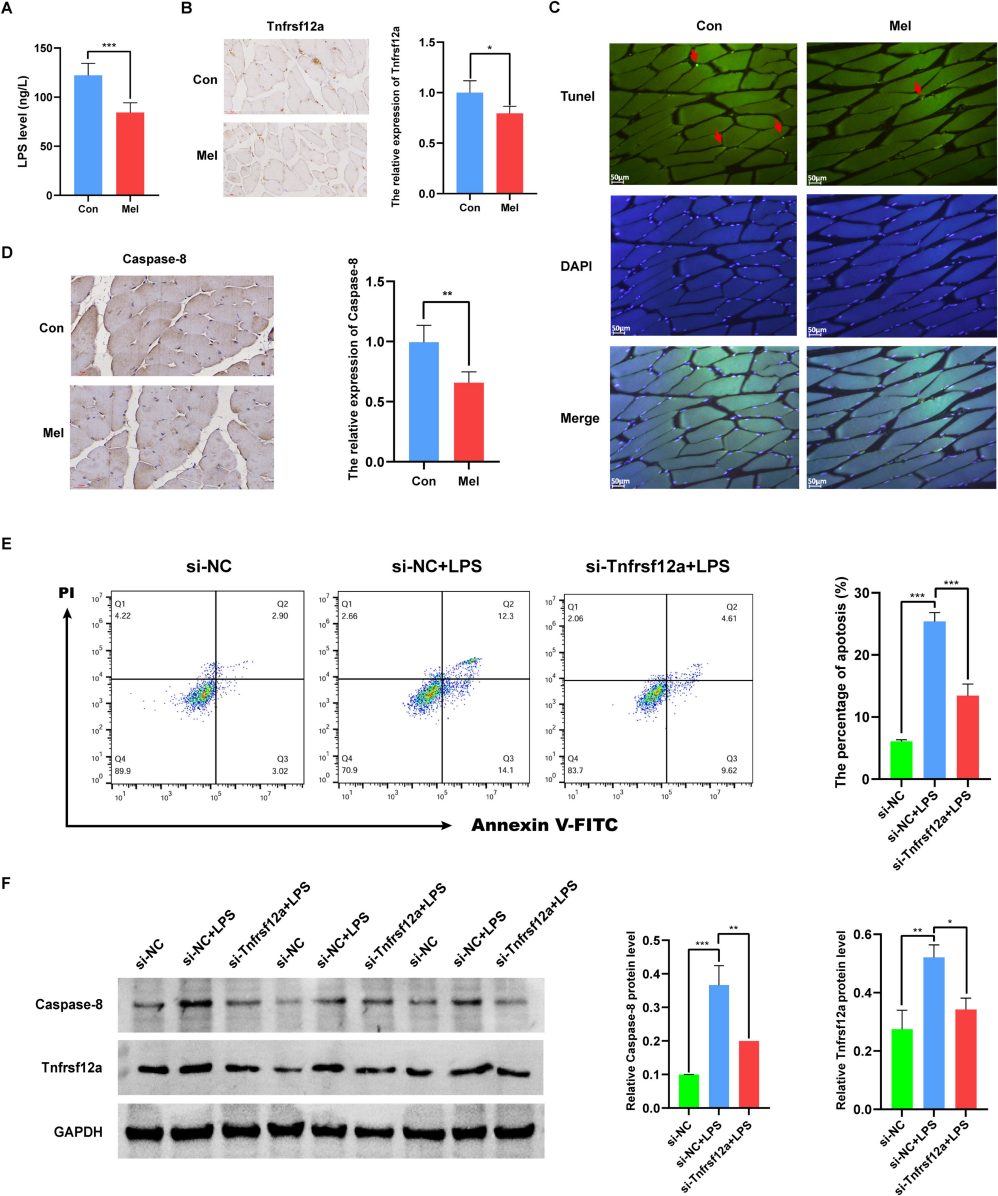
The LPS‐induced Tnfrsf12a/caspase‐8 signalling pathway is involved in the mechanism by which melatonin alleviates age‐associated sarcopenia via the gut–muscle axis. (A) The serum level of LPS; (B) the immunohistochemical staining of Tnfrsf12a in gastrocnemius of aged mice; (C) TUNEL analysis of apoptosis in gastrocnemius; (D) the immunohistochemical staining of caspase‐8 in gastrocnemius of aged mice; (E) the percentages of apoptotic C2C12 myoblasts; (F) the protein expression of Tnfrsf12a and caspase‐8 in C2C12 myoblasts. *p < 0.05, **p < 0.01, ***p < 0.001.

As previously mentioned, LPS biosynthesis was predicted as one of the main significantly different microbiota functions (Table S2). We then checked whether serum LPS showed differences in aged mice with or without melatonin administration, and if so, whether it was related to age‐related DEGs in skeletal muscle. As shown in Figure 4A, we observed significantly decreased serum LPS levels in aged mice with melatonin administration. To further explore the effect of changes in serum LPS on the skeletal muscle ageing, we firstly looked for DEGs specifically in relation to ageing and melatonin. Of the 33 DEGs associated with melatonin administration and the 2376 DEGs associated with muscle ageing, 12 DEGs were associated with both and identified as the potential melatonin targets for the therapy of age‐related sarcopenia (Figure S2, Table S4). Subsequently, the Pearson correlation analysis between 12 DEGs and LPS was conducted, and 11 DEGs related to serum LPS were suggested to be of a great significance (Table S5). The top association was observed for Tnfrsf12a (Figure S3). And the Tnfrsf12a‐positive area indicated by immunohistochemistry was significantly lower in the Mel group (Figure 4B). We also consistently observed that the mRNA level of Tnfrsf12a was negatively correlated with the average CSA of muscle fibre sizes *(*Figure S4). These results suggest that LPS‐related Tnfrsf12a may be involved in the mechanism of melatonin ameliorating age‐related sarcopenia. As shown in Figure 3B,C, the regulation of extrinsic apoptotic signalling pathways was predicted as the main biological function of melatonin in the treatment of sarcopenia, and the noncanonical NF‐κB pathway mediated by TNF receptor superfamily members might be the potential mechanism. Caspase‐8, as the initiator caspase of extrinsic apoptosis, has been suggested as an additional consideration when investigating the downstream noncanonical NF‐κB signalling events of TWEAK‐Tnfrsf12a activation (Supporting Information: Reference S6). We further explored the cell apoptosis and expression of caspase‐8 in skeletal muscle. As expected, both were significantly decreased after melatonin treatment (Figure 4C,D). These findings indicate that the melatonin‐related modification of gut microbiota ameliorating sarcopenia may be connected to the LPS‐related Tnfrsf12a/caspase‐8 signalling pathway. To verify the conclusion, we further conducted in vitro experiments by stimulating C2C12 myoblasts with LPS. As presented in Figure 4E,F, LPS treatment significantly increased the percentage of apoptotic cells and induced the protein expression of Tnfrsf12a and Caspase‐8 in C2C12 myoblasts. Nevertheless, Tnfrsf12a gene silencing by siRNA gave rise to a significant decrease in LPS‐triggered apoptosis, as well as Caspase‐8 expressions compared with that in the cells transfect with si‐NC (Figure 4E,F).

### 3.5 Melatonin Alters the Blood Metabolic Profiling

**FIGURE 5:**
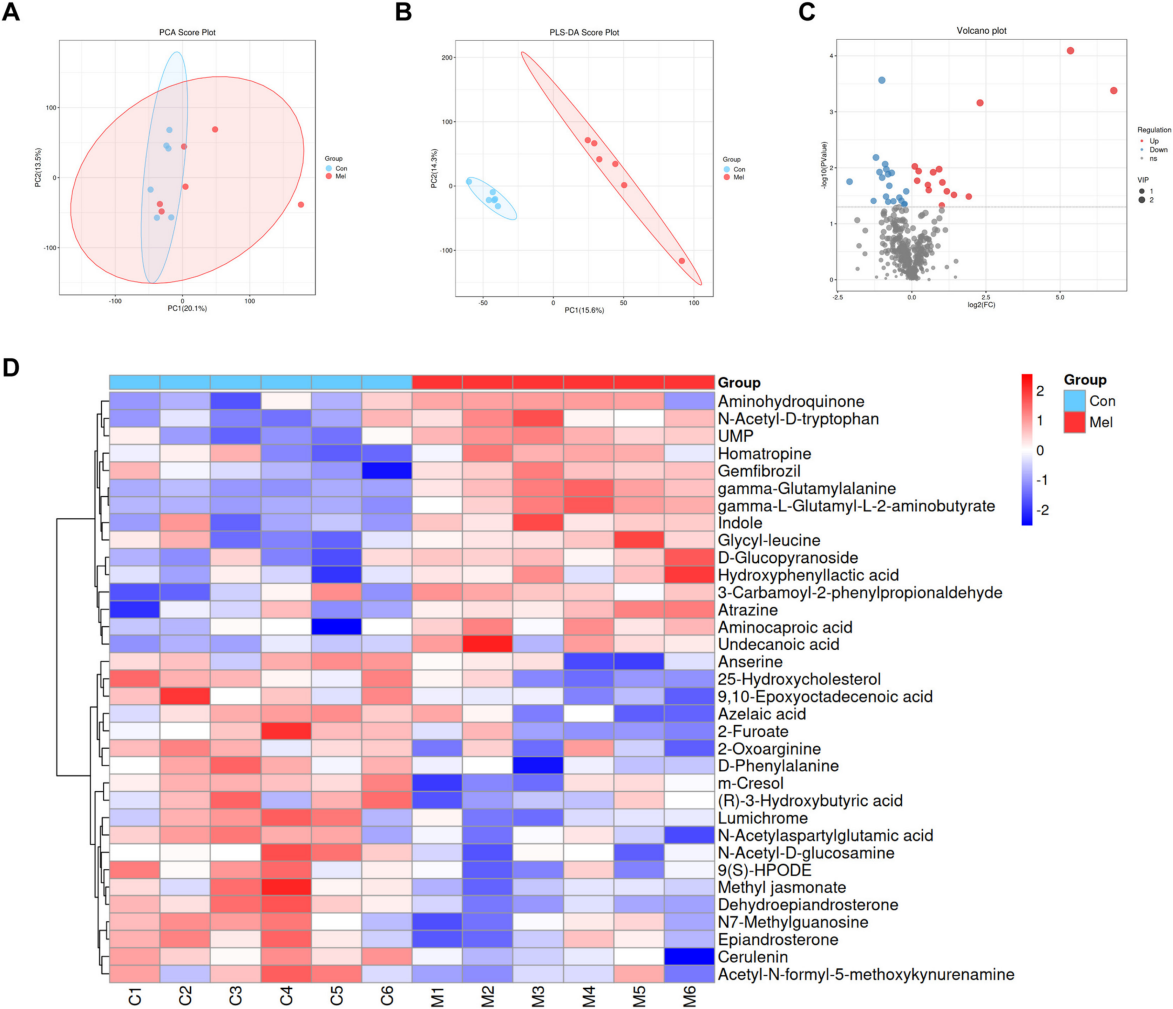
Melatonin intake affects serum metabolomic profiles in aged mice. (A) Principal component analysis (PCA) plots of Con and Mel groups; (B) partial least‐squares‐discriminant analysis (PLS‐DA) plots of Con and Mel groups; (C) the volcano plots of melatonin Con and Mel groups; (D) hierarchical cluster analysis of serum differential metabolites between Con and Mel groups. *n* = 6.

Apart from LPS biosynthesis, several pathways of metabolites displayed significant differences in predicting microbiota functions (Table S2). Therefore, we hypothesized that melatonin could alter the serum metabolites of mice, including microbiota‐related metabolites alleviating age‐associated sarcopenia. The unsupervised principal component analysis (PCA) and partial least squares‐discriminate analysis (PLS‐DA) revealed the significant discrimination between the Mel and control groups (Figure 5A,B), indicating the reliable differences of blood metabolome. Based on the variable importance on projection (VIP) > 1.5 and *p* < 0.05, we identified 34 significantly altered metabolites (Figure 5C, Table S6). A heatmap of the comparative analysis exhibited the expression levels of the metabolites (Figure 5D). Compared with the control, melatonin significantly increased the content of gamma‐glutamylalanine, gamma‐L‐glutamyl‐L‐2‐aminobutyrate, UMP, D‐glucopyranoside, N‐acetyl‐D‐tryptophan, indole and other metabolites. Taken together, melatonin intake is associated with significant changes in the blood metabolome of aged mice.

### 3.6 The Microbiome Contributes to Muscle Transcriptomic Changes Through Metabolites

**FIGURE 6:**
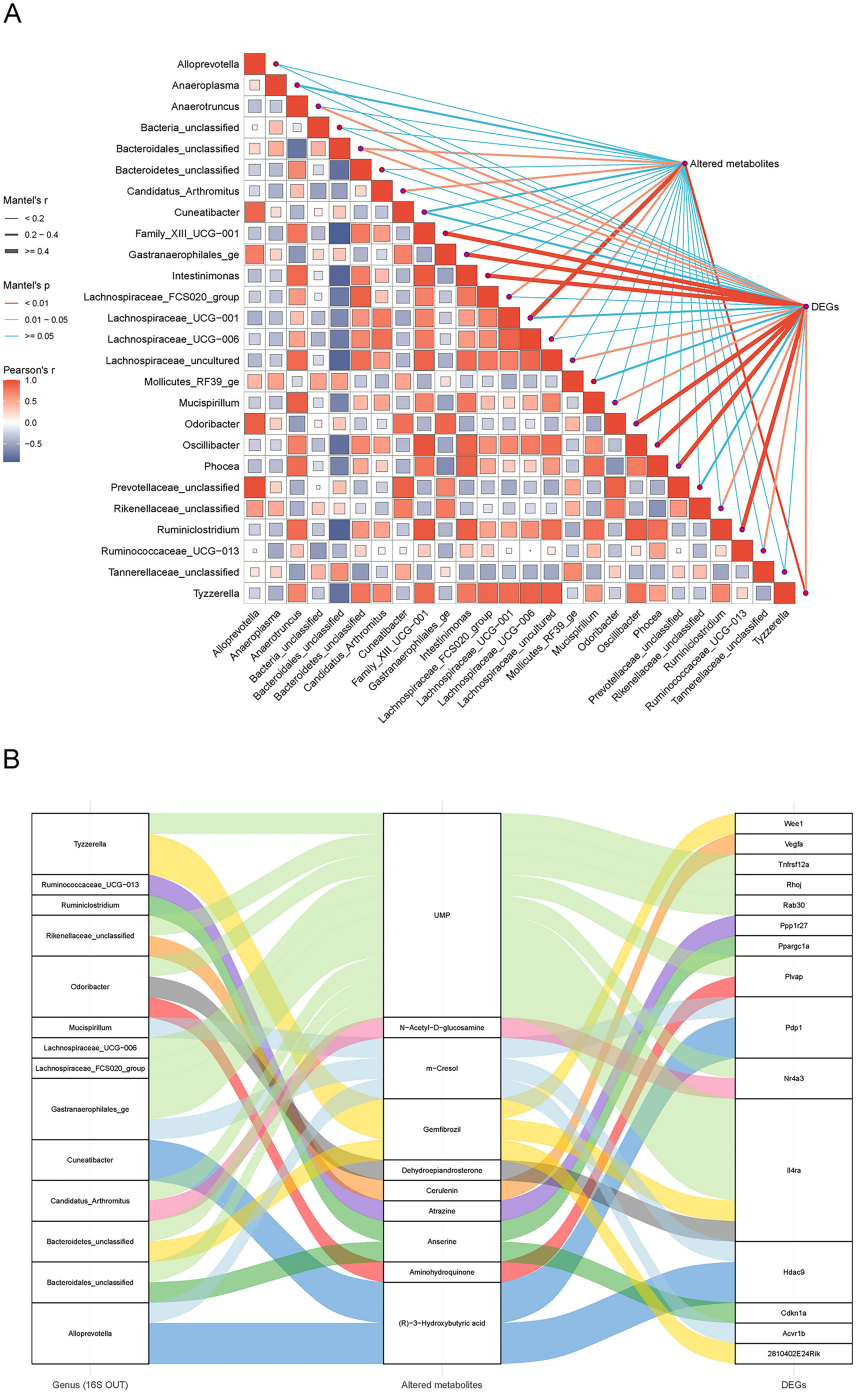
The mediation role of metabolites in the gut microbiota–skeletal muscle axis. (A) The correlations among the gut microbiome, serum metabolites and muscle genes; (B) Sankey diagram showing the significant mediation effects of serum metabolites in the gut microbiota–skeletal muscle axis.

To further understand the potential mechanisms by which the changed gut microbiota facilitated related pathophysiology and the role of metabolites in gut–muscle communications, we first checked whether the changed gut microbiota was associated with the altered metabolites and DEGs using the Pearson correlation analysis and Mantel‐test analysis. As shown in Figure 6A, there existed a vast number of significant correlations among them, implying that the unique microbial structure after melatonin treatment played an important role in metabolism and muscle transcriptome. For the role of metabolites, we hypothesized that metabolites were involved in the gut–muscle communication and could mediate the microbial impact on DEGs. Subsequently, we applied mediation analyses and observed 27 mediation linkages (Figure 6B; Table S7). Most of these linkages were related to microbial impact on DEGs via UMP and (R)‐3‐hydroxybutyric acid. These findings suggest that metabolites may serve as important intermediates in the gut–muscle communication, substantially alleviating the development of age‐related sarcopenia.

## 4 Discussion

**FIGURE 7:**
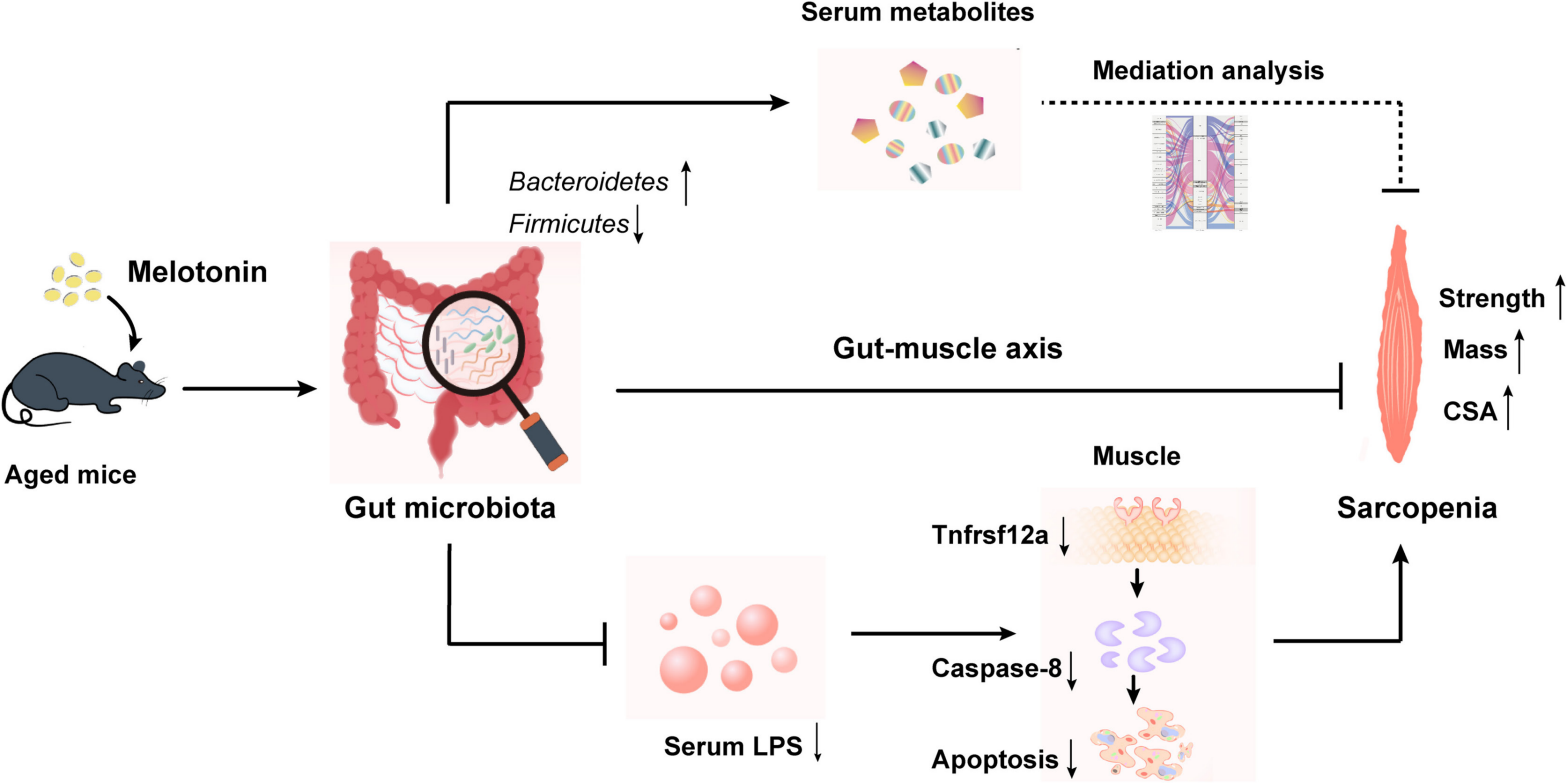
The mechanism diagram of this study for explaining the effect of melatonin on age‐related sarcopenia through the gut–muscle axis.

In this study, we demonstrated that the melatonin supplementation could ameliorate sarcopenia, including ageing‐elicited declines in muscle strength, muscle mass and CSA of the gastrocnemius muscle fibres. In addition, melatonin significantly changed the composition of the gut microbiota. However, the key to revealing the role of melatonin‐modified gut microbiota in sarcopenia is to answer how the gut microbiota achieves crosstalk between itself and distant skeletal muscles. Functional predictions of altered gut microbiota gave us important clues about this. And we found factors (serum LPS and metabolites) released to skeletal muscle might play a significant role in the gut–muscle crosstalk. To verify this hypothesis, we therefore detected the level of serum LPS and characterized the serum metabolite profiles. As expected, significant differences in LPS levels and serum metabolites were observed between the control and melatonin groups. In particular, we found that the gut microbiota‐modulated LPS could alleviate age‐associated sarcopenia by regulating the Tnfrsf12a/caspase‐8 signalling pathway via integrated multiomics analyses and in vivo and in vitro experiments. Finally, mediation models revealed the causal relationships between gut microbiomes, serum metabolites and genes of skeletal muscle, and provided putative mechanistic insights into the gut microbiome altered by melatonin in sarcopenia, indicating that serum metabolites could work as intermediates in the gut–muscle communication (Figure 7).

Endogenous melatonin decreases in an age‐dependent manner. And melatonin administration can counteract many senescence‐related changes in aged animals, such as age‐related osteoporosis, age‐related cardiac alterations and abnormalities and ovarian ageing (Supporting Information: References S7–S9). This current available evidence suggests its good application prospects as a dietary supplement to prevent and treat ageing and age‐related diseases. Our study demonstrated that oral administration of melatonin contained in drinking water improved age‐related sarcopenia in naturally ageing mice. This result complements a previous report that melatonin administration can conserve the normal muscular architecture, weight, fibre number and activity in the old‐age muscle, which provides the new proof for its potential utility in the antiageing aspect, especially the treatment of age‐related sarcopenia [6].

So far, the only proven antiageing method is diet restriction, and one of its mechanisms is probably mediated by an increase in melatonin in the gastrointestinal tract [17]. Then, how does the elevated melatonin delay the process of ageing? Several bacterial sequences were found with high similarity to the melatonin binding sites in the MT1 and MT2 receptors. There is evidence that at least one type of gut bacteria expressing similar sequences was specifically affected by concentrations of melatonin, not by other indoles, which related melatonin to gut microbiome as an exocrine signal [18]. And these findings can partially account for the effect of melatonin on the gut–organ axis, thus inspiring our research on the potential role of melatonin in the gut–muscle axis involved in age‐related sarcopenia. There is no doubt that melatonin can affect muscles through various mechanisms [19]. So, our study does not claim that the effects of melatonin on age‐related sarcopenia are entirely mediated by the gut microbiota. In this study, we observed that melatonin altered the intestinal microbial species composition in aged mice, with the increased proportion of *Bacteroidetes* and decreased proportion of *Firmicutes*. The increased F/B ratio was observed in aged mice, indicating dysbiosis, which is widely believed to have an important impact on systemic inflammation [20, 21]. And inflammation has been proven to be involved in the pathogenesis of sarcopenia and muscle modulation of microbiome [22]. Thus, the changed gut microbiota might alleviate age‐related sarcopenia through inflammation in the melatonin group. In addition, further analyses at genus levels revealed that melatonin treatment could improve the relative abundance of *Odoribacter* in aged mice. Interestingly, *Odoribacter* is one butyrate‐producing bacteria and has the anti‐inflammatory capacity by inhibiting the release of inflammatory cytokines [23, 24]. Not only gut dysbiosis, ageing can also contribute to intestinal barrier dysfunction that negatively affects muscle strength and function [25]. And *Odoribacter‐* generated butyrate can enhance the integrity of the gut barrier by upregulating the expression of tight junction proteins [23]. Moreover, it has been reported that the relative abundance of *Rikenellaceae* is significantly decreased with age, but was increased after melatonin treatment in the present study [26]. An elevated abundance of *Rikenellaceae* has been demonstrated to be related to the reduced visceral adipose tissue and healthier metabolic profile and could be one potential marker of healthy ageing [27]. These results confirm that melatonin can modulate the intestinal flora in aged mice with sarcopenia, which also provides evidence for its use as a probiotic agent. Indeed, current research has shown that age‐related sarcopenia may benefit, at least in part, from the probiotic interventions aiming to modulate the gut microbiota [28].

Because the association between the modulated gut microbiota and sarcopenia was observed, we further investigated how the gut microbiota drove the relevant pathophysiology of sarcopenia. Increasing evidence has demonstrated that LPS production modulated by the gut microbiota could affect the host skeletal muscle and might contribute to the occurrence of sarcopenia [29]. We observed that melatonin could downregulate the level of LPS through modulating the gut microbiota. More recently, Wang et al. demonstrated the same results in mice with cognitive impairment induced by sleep deprivation [15]. Age‐related modifications in the gut microbiota can promote intestinal mucosa permeability. As mentioned above, *Odoribacter* could enhance the integrity of the gut barrier, thus decreasing the entry of LPS into circulation, which may partly account for the effect of melatonin on the serum LPS level through the gut microbiota. Then, how does the decreased serum LPS exert its effect on skeletal muscle? Using multiomics methods, we found that the expression of Tnfrsf12a, significantly downregulated by melatonin, is most correlated with the level of serum LPS. It was reported that the level of Tnfrsf12a was increased in skeletal muscle of aged mice and the genetic ablation of Tnfrsf12a could blunt age‐related muscle atrophy [30]. Moreover, LPS could induce the expression of Tnfrsf12a in C2C12 cells, which was consistent with the finding in porcine small intestinal epithelial cells [31]. These findings provide evidence for that serum LPS may play a meaningful role in the gut–muscle axis by regulating the expression of Tnfrsf12a in skeletal muscle. Combining bioinformatics analyses and experiment validation, we further found that LPS could elicit apoptosis in skeletal muscle by regulating the Tnfrsf12a/caspase‐8 pathway. Apoptosis is known to play an important role during muscle ageing. And the expression of caspase‐8 involved in apoptotic signalling pathways was increased with ageing in skeletal muscle and correlated with the progression of sarcopenia [32]. Additionally, Tnfrsf12a has been confirmed to positively regulate the expression of caspase‐8 upon TWEAK stimulation [33]. Thus, above results suggest that the LPS‐activated Tnfrsf12a/caspase‐8 pathway is involved in the mechanism by which melatonin relieves age‐related sarcopenia through the gut–muscle axis.

In addition, we observed that melatonin could alter blood metabolites involved in inflammation and oxidative stress, such as indole, gamma‐glutamylalanine, gamma‐L‐glutamyl‐L‐2‐aminobutyrate and D‐glucopyranoside [34, 35]. Among all, serum metabolites (R)‐3‐hydroxybutyric acid, one of certain biomarkers for muscle mass loss in HBV‐related cirrhosis patients, was decreased in aged mice with melatonin [36]. Notably, there exists causality that metabolites produced by the gut microbiota can enter the systemic circulation and signal to distant organs, thereby influencing host health [37]. To date, however, most cross‐sectional studies just reveal the connections between the gut microbiota, metabolites and diseases, without inferring potential causal relationships. Thanks to progress in computational biology and bioinformatics, we have opportunities to obtain insights into the complex network. The mediation analysis, as an important statistical tool for understanding the mechanisms of exposure‐mediator‐outcome effects, is recommended for causal interpretation of the effect estimates in observational research [16]. By coupling multiomics bioinformatics with mediation analyses, Chen et al. revealed that the microbiome can contribute to host phenotypic changes through metabolites [38]. We also observed that metabolites could mediate the melatonin‐related microbial impact on skeletal muscle. Notably, the associations of metabolites with the gut microbiome modified by melatonin were significantly enriched for UMP and (R)‐3‐hydroxybutyric acid. Besides (R)‐3‐hydroxybutyric acid mentioned above, UMP has been reported to have an impact on skeletal muscle [39]. Altogether, by combining multiomics bioinformatics with mediation analyses, our findings further reveal the functional potential of the melatonin‐related modifications in the gut microbiota for sarcopenia from another perspective, namely the putative causality for the gut microbiome‐metabolites‐muscle axis.

Of note, there exist several limitations in our study. Our findings provide reasonable evidence for the effect of melatonin on age‐related sarcopenia through the gut–muscle axis. Nevertheless, this evidence cannot fully demonstrate the direct relationship between the melatonin‐modified gut microbiota and muscles. A faecal transplantation experiment may be a good choice for explaining the direct relationship. But the gut microbiota composition was shaped by a daily melatonin treatment. As mentioned above, melatonin can act as an exocrine signal to the gut microbiome. Without melatonin stimulation, the microbial community interactions may change, leading to the inability to reproduce the effect of melatonin‐related modifications in the gut microbiota. In addition, we preliminarily indicated the most important intermediates in the gut–muscle communication by coupling multiomics bioinformatics with mediation analyses. The experimental validation is still needed for this observation of causal relationships.

## Ethics Statement

This animal study was reviewed and approved by the Animal Ethics Committee of the first hospital of Lanzhou University (Approval No. LDYYLL2022‐41) and has therefore been performed in accordance with the ethical standards laid down in the 1964 Declaration of Helsinki and its later amendments.

## Conflicts of Interest

The authors declare no conflicts of interest.

## Supporting information


**Data S1** Supplementary Information.

**Data S2** Supplementary Methods.

**Data S3** Supplementary Reference.

**Data S4** Supplementary Figure Legend.

**Figure S1** Effect of melatonin on variation of the gut microbiome composition. (A) The genus‐level ACE alpha‐diversity index in Con and Mel groups; (B) genus‐level Shannon alpha‐diversity index in Con and Mel groups; (C) intestinal microbe relative abundance at the genus level; Microbial co‐occurrence network in Con group (D) and Mel group (E).

**Figure S2** Venn plot of the number of DEGs that were associated with melatonin administration and muscle aging.

**Figure S3** Pearson correlation analysis for the level of Tnfrsf12a mRNA in gastrocnemius and serum level of LPS.

**Figure S4** Pearson correlation analysis for CSA of fibre sizes and the level of Tnfrsf12a mRNA in gastrocnemius (Each dot represents a gastrocnemius).

**Table S1** The list of abundance of intestinal microbe at the genus levels.

**Table S2** The functions predicted with significance in KEGG pathways.

**Table S3** The list of differentially expressed genes in skeletal muscle of aged mice.

**Table S4** The list of differentially expressed genes in skeletal muscle between young and aged mice.

**Table S5** Pearson correlation analysis for LPS level and 12 DEGs.

**Table S6** The list of significantly altered metabolites by melatonin.

**Table S7** The results of mediation analysis.

## Data Availability

The transcriptomics raw data used in this study are available in a public database (https://data.mendeley.com/preview/7h29rcnyvb?a=15d1c462‐f10f‐4c43‐b7de‐64101a58e581).
