# Supplementary material for: Melatonin Ameliorates Age‐Related Sarcopenia via the Gut–Muscle Axis Mediated by Serum Lipopolysaccharide and Metabolites
Source: J Cachexia Sarcopenia Muscle. 2025 Feb 3;16(1):e13722. doi: 10.1002/jcsm.13722 (PMC11790590; doi:10.1002/jcsm.13722)
Supplement: Supplementary file 1 — Data S1 Supplementary Information. [file JCSM-16-e13722-s010.docx]

**Feed Description**

Product ingredient: corn, soybean meal, fish meal, flour, yeast powder, vegetable oil, salt, various vitamins, mineral elements and so on.

Nutritional information:

| Nutrient | Content |
| --- | --- |
| Moisture content | ≤10% |
| Crude protein | ≥18% |
| Crude fat | ≥4% |
| Crude fiber | ≤5% |
| Crude ash | ≤8% |
| Calcium | 1.0 ~ 1.8% |
| Total phosphorus | 0.6 ~1.2% |
