# Supplementary material for: Melatonin Ameliorates Age‐Related Sarcopenia via the Gut–Muscle Axis Mediated by Serum Lipopolysaccharide and Metabolites
Source: J Cachexia Sarcopenia Muscle. 2025 Feb 3;16(1):e13722. doi: 10.1002/jcsm.13722 (PMC11790590; doi:10.1002/jcsm.13722)
Supplement: Supplementary file 2 — Data S2 Supplementary Methods. [file JCSM-16-e13722-s006.docx]

**Methods S1** Total RNA was extracted from skeletal muscle using Trizol reagent (Ambion, USA) following by the manufacturer’s protocol and quantified through spectrophotometry using NanoDrop One/OneC (Thermo Fisher Scientific, USA). RNA concentration was mesuared using Life Invitrogen Qubit 3.0 (Thermo Fisher Scientific, USA) and RNA integrity was determined by Agilent 4200 TapeStation (Agilent, USA). After verifying RNA quality, cDNA library was constructed by capturing mRNA, synthesizing the double-stranded cDNA, screening and amplified cDNA. The obtained cDNA library for each sample was sequenced on the Illumina PE150 platform. The raw data was processed to filter the low-quality sequences using fastp software. And clean reads were obtained and mapped against to the reference genome by HISAT2 software. BCFtools was performed to sort and filter duplicate reads in generated BAM files. The read counts for all annotated genes were generated using HTSeq software. The differentially expressed genes (DEGs) between the different groups were screened with the thresholds of absolute fold change >1.5 and FDR <0.05. To explore the underlying mechanism, we conducted the Gene Ontology (GO) and Reactome pathways analyses using the "clusterProfiler" R package.

**Methods S2** Microbial DNA was extracted from each fecal samples using the QIAamp DNA Stool Mini Kit (Qiagen, Germany) according to the manufacturer’s protocol. The V3–V4 region of 16S rRNA gene was amplified by PCR using primers 341F (5’- CCTACGGGNGGCWGCAG-3’) and 785R (5’- GACTACHVGGGTATCTAATCC-3’). Then, the amplicons were extracted from 2% agarose gels and purified using QIA quick Gel Extraction Kit (Qiagen, Germany). After quality assessment by examining DNA concentration and length distribution, the qualified PCR products were quantified using KAPA Library Quantification Kit (KAPA Biosystems, USA) and then pooled at equimolar ratios. Finally, the paired-end sequencing (2×250) of constructed DNA library was performed on the Illumina Novaseq 6000 platform (San Diego, USA). From the raw genomics sequencing data, pair-end reads were merged using “fastq_mergepairs” command in [vsearch](https://github.com/torognes/vsearch) software. To obtain clean data, the low-quality reads were then further discarded based on “fastq_filter command”. And the clean reads based on sequences, greater than 97% similarity, were clustered into different Operational Taxonomic Units (OTUs) through the UPARSE pipeline. Microbial taxonomic profiles from Phylum to Genus were determined by mapping each OUT representative gene sequence to the SILVA Database using the “classify.seqs” command in mothur software with confidence threshold values more than 0.8. Alpha-diversity analysis was performed by calculating the index of community richness (ACE index) and index of community diversity (Shannon index). Beta diversity was assessed using principal coordinate analysis (PCA). And the statistical significance of differences in beta-diversity was assessed by analysis of similarities (ANOSIM). Linear discriminant analysis (LDA) effect size (LEfSe) analysis was conducted to identify the biological markers with statistical differences between groups. PICRUST2 database was used to predict the functions of the microbiota.

**Methods S3** The separation of metabolic extracts was carried out using UHPLC System, equipped with a UPLC BEH Amide column. The mobile phase consisted of 25 mmol/L ammonium acetate and 25 mmol/L ammonia hydroxide in water as solvent A and acetonitrile as solvent B in gradient elution mode. The MS/MS spectra was obtained by using a triple time-of-flight (TOF) mass spectrometer (AB Sciex) during an LC/MS experiment. When collecting and triggering the acquisition of MS/MS spectra, the acquisition software (Analyst TF 1.7, AB Sciex) continuously evaluated the full scan survey MS data based on pre-selected criteria. The raw data generated by LC-MS were converted to mzXML format by MSConvert in ProteoWizard software package (v3.0.8789) and processed using XCMS software to perform peak integration, calibration, and quantification for each metabolite. Metabolomic features were annotated to metabolites with accuracy mass (< 30 ppm) and MS/MS data which were matched with HMDB (http://www.hmdb.ca), massbank (http://www.massbank.jp/), LipidMaps (<http://www.lipidmaps.org>), mzcloud (https://www.mzcloud.org) and KEGG (http://www.genome.jp/kegg/). After normalization, we further excluded metabolites with the relative standard deviations (RSDs) larger than 30 % in QC samples. The Ropls software was used for principal component analysis (PCA) and orthogonal partial least-square discriminant analysis (PLS-DA) to provide information on the metabolites that influence clustering of the samples. The P value, Variable importance projection (VIP) produced by partial least-square discriminant analysis (OPLS-DA), and fold change (FC) were applied to discover the contributable-variable for classification. Finally, P＜0.05 and VIP＞1 were set as a statistical threshold for discriminating significantly differential metabolites.
