# Supplementary material for: Melatonin Ameliorates Age‐Related Sarcopenia via the Gut–Muscle Axis Mediated by Serum Lipopolysaccharide and Metabolites
Source: J Cachexia Sarcopenia Muscle. 2025 Feb 3;16(1):e13722. doi: 10.1002/jcsm.13722 (PMC11790590; doi:10.1002/jcsm.13722)
Supplement: Supplementary file 3 — Data S3 Supplementary Reference. [file JCSM-16-e13722-s011.docx]

**References**

S1. Godfrey S, Iversen HK, West AS. Melatonin profile in healthy, elderly subjects - A systematic literature review. *Chronobiology international* 2022;**39(4)**:476-492.

S2. Luo F, Sandhu AF, Rungratanawanich W, Williams GE, Akbar M, Zhou S,et al. Melatonin and Autophagy in Aging-Related Neurodegenerative Diseases. *International journal of molecular sciences* 2020;**21(19)**:7174.

S3. Tilg H, Adolph TE, Trauner M. Gut-liver axis: Pathophysiological concepts and clinical implications. *Cell metabolism* 2022;**34(11)**:1700-1718.

S4. Agirman G, Yu KB, Hsiao EY. Signaling inflammation across the gut-brain axis. *Science (New York, NY)* 2021;**374(6571)**:1087-1092.

S5. Cao YY, Wang Z, Yu T, Zhang Y, Wang ZH, Lu ZM, et al. Sepsis induces muscle atrophy by inhibiting proliferation and promoting apoptosis via PLK1-AKT signalling. *J Cell Mol Med* 2021;**25(20)**:9724-9739.

S6. Pascoe AL, Johnston AJ, Murphy RM. Controversies in TWEAK-Fn14 signaling in skeletal muscle atrophy and regeneration. *Cell Mol Life Sci* 2020;**77(17)**:3369-3381.

S7. Igarashi-Migitaka J, Seki A, Ikegame M, Honda M, Sekiguchi T, Mishima H, et al. Oral administration of melatonin contained in drinking water increased bone strength in naturally aged mice. *Acta histochemica* 2020;**122(6)**:151596.

S8. Favero G, Franceschetti L, Buffoli B, Moghadasian MH, Reiter RJ, Rodella LF, et al. Melatonin: Protection against age-related cardiac pathology. *Ageing research reviews* 2017;**35**:336-349.

S9. Tamura H, Kawamoto M, Sato S, Tamura I, Maekawa R, Taketani T, et al. Long-term melatonin treatment delays ovarian aging. *Journal of pineal research* 2017; **62(2)**.
