# Supplementary material for: Melatonin Ameliorates Age‐Related Sarcopenia via the Gut–Muscle Axis Mediated by Serum Lipopolysaccharide and Metabolites
Source: J Cachexia Sarcopenia Muscle. 2025 Feb 3;16(1):e13722. doi: 10.1002/jcsm.13722 (PMC11790590; doi:10.1002/jcsm.13722)
Supplement: Supplementary file 4 — Data S4 Supplementary Figure Legend. [file JCSM-16-e13722-s003.docx]

Figure S1 Effect of melatonin on variation of the gut microbiome composition. (A) The genus-level ACE alpha-diversity index in Con and Mel groups; (B) genus-level Shannon alpha-diversity index in Con and Mel groups; (C) intestinal microbe relative abundance at the genus level; Microbial co-occurrence network in Con group (D) and Mel group (E).

Figure S2 Venn plot of the number of DEGs that were associated with melatonin administration and muscle aging.

Figure S3 Pearson correlation analysis for the level of Tnfrsf12a mRNA in gastrocnemius and serum level of LPS.

Figure S4 Pearson correlation analysis for CSA of fiber sizes and the level of Tnfrsf12a mRNA in gastrocnemius (Each dot represents a gastrocnemius).
