# Supplementary figures and images for: Melatonin Ameliorates Age‐Related Sarcopenia via the Gut–Muscle Axis Mediated by Serum Lipopolysaccharide and Metabolites
Source: J Cachexia Sarcopenia Muscle. 2025 Feb 3;16(1):e13722. doi: 10.1002/jcsm.13722 (PMC11790590; doi:10.1002/jcsm.13722)

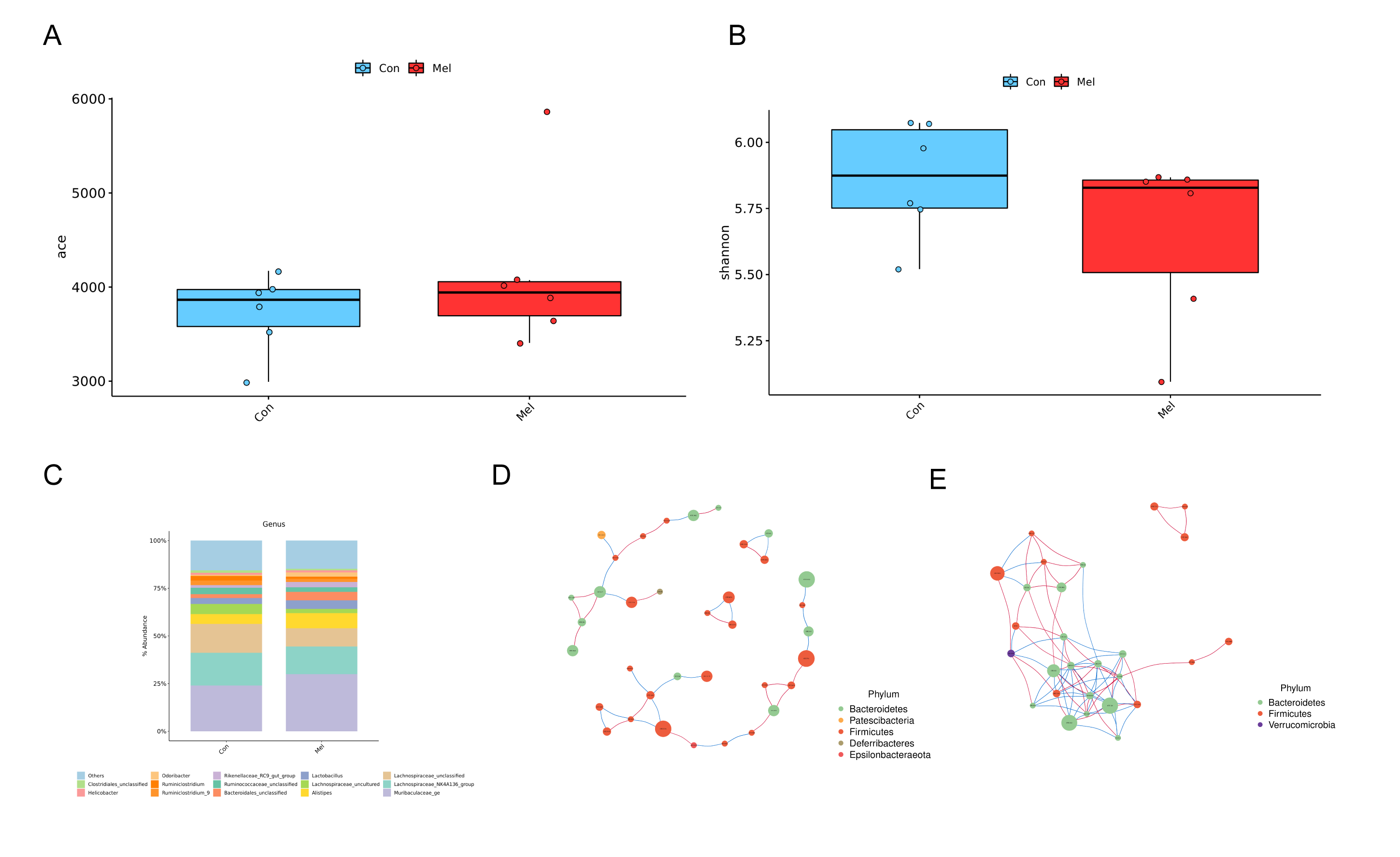

Supplement: Supplementary file 5 — Figure S1 Effect of melatonin on variation of the gut microbiome composition. (A) The genus‐level ACE alpha‐diversity index in Con and Mel groups; (B) genus‐level Shannon alpha‐diversity index in Con and Mel groups; (C) intestinal microbe relative abundance at the genus level; Microbial co‐occurrence network in Con group (D) and Mel group (E). [file JCSM-16-e13722-s013.tif]

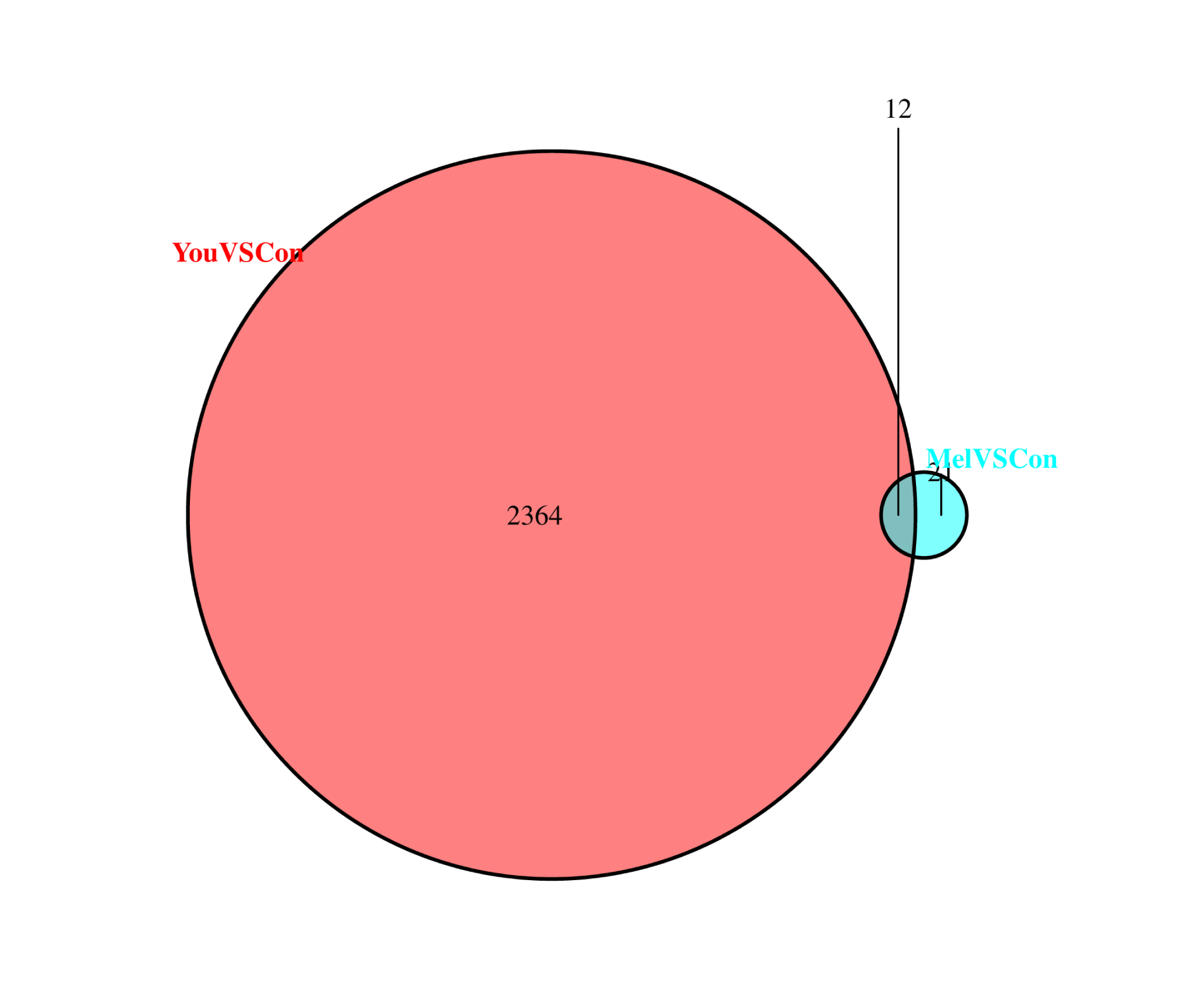

Supplement: Supplementary file 6 — Figure S2 Venn plot of the number of DEGs that were associated with melatonin administration and muscle aging. [file JCSM-16-e13722-s001.tif]

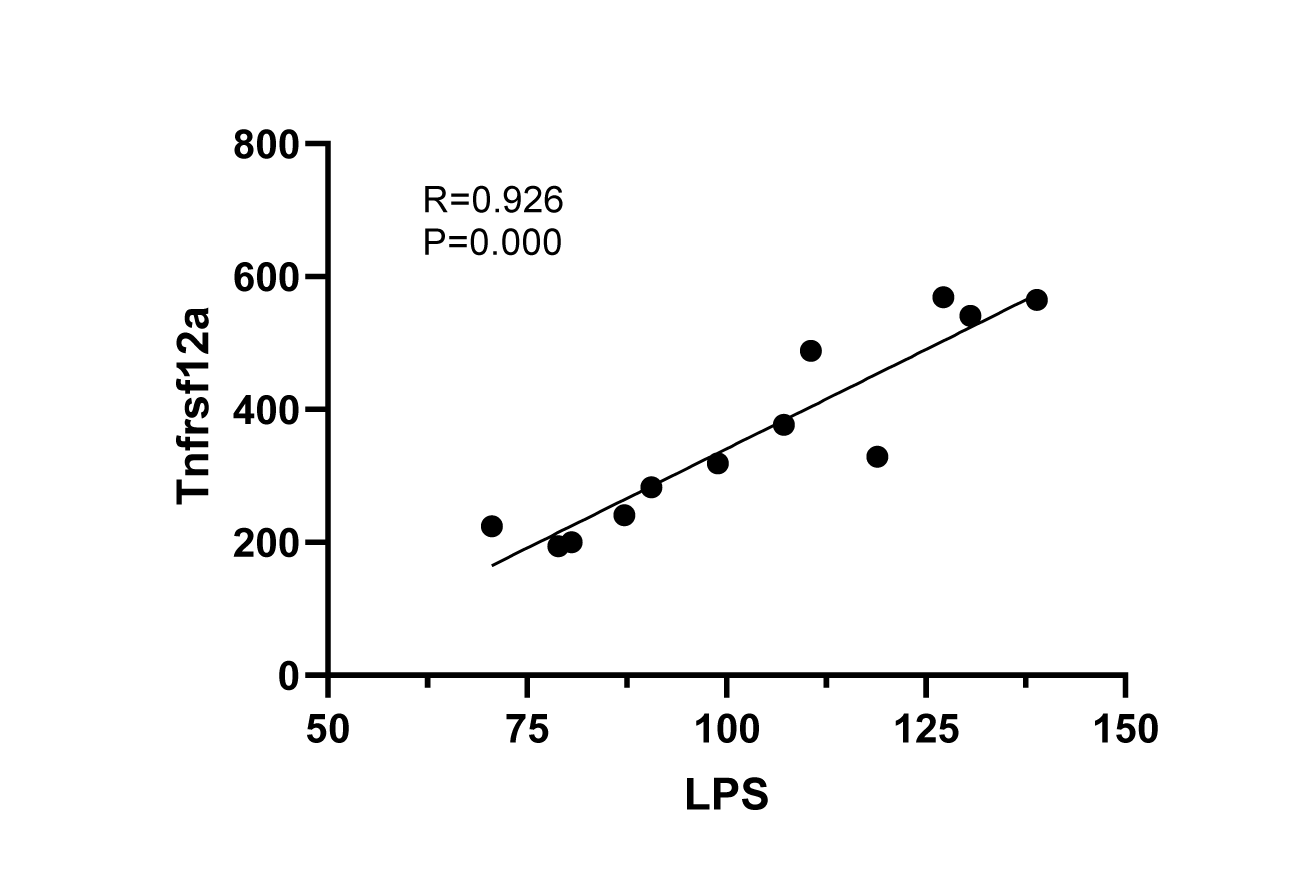

Supplement: Supplementary file 7 — Figure S3 Pearson correlation analysis for the level of Tnfrsf12a mRNA in gastrocnemius and serum level of LPS. [file JCSM-16-e13722-s004.tif]

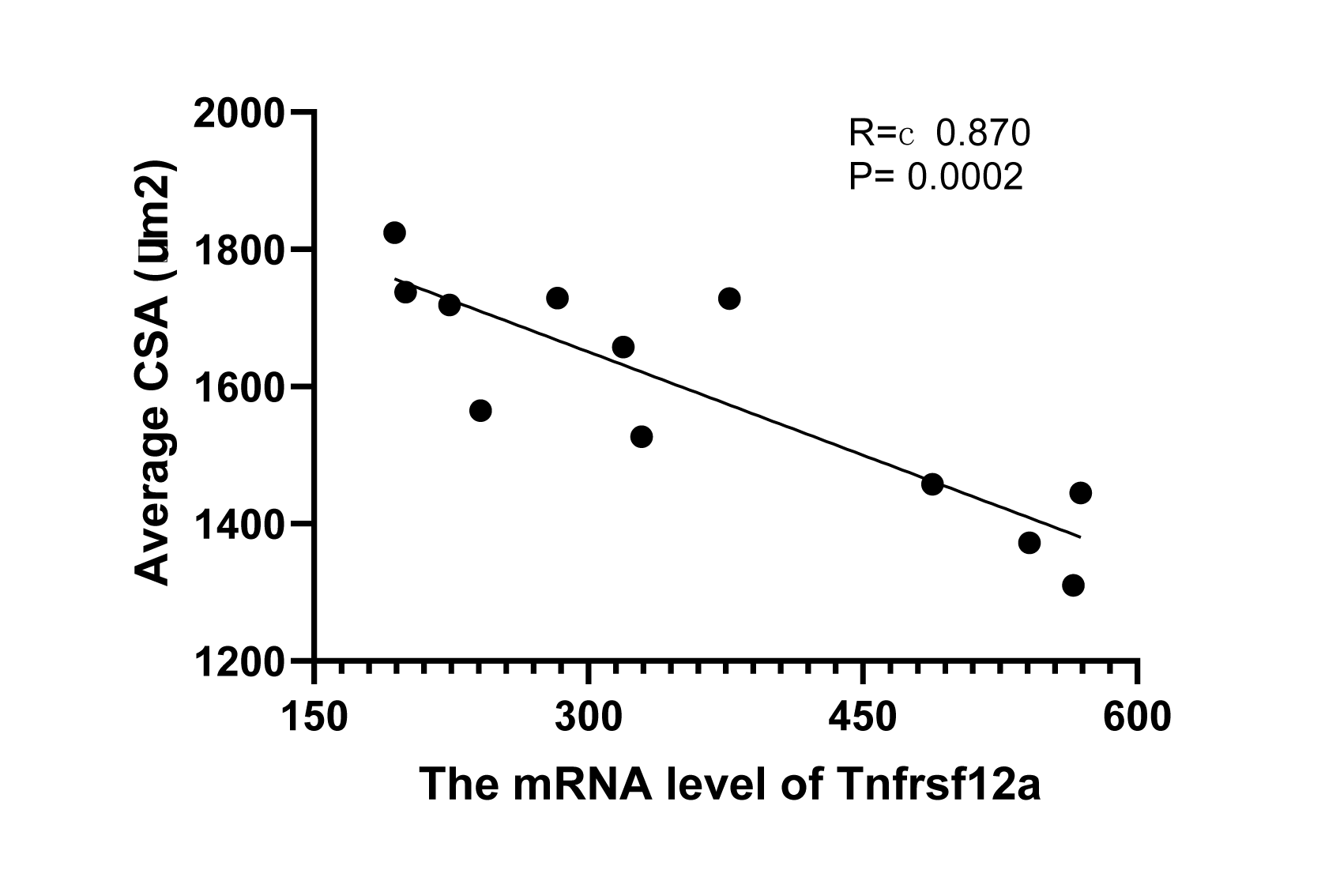

Supplement: Supplementary file 8 — Figure S4 Pearson correlation analysis for CSA of fibre sizes and the level of Tnfrsf12a mRNA in gastrocnemius (Each dot represents a gastrocnemius). [file JCSM-16-e13722-s009.tif]
